# An Online Database for Informing Ecological Network Models: http://kelpforest.ucsc.edu

**DOI:** 10.1371/journal.pone.0109356

**Published:** 2014-10-24

**Authors:** Rodrigo Beas-Luna, Mark Novak, Mark H. Carr, Martin T. Tinker, August Black, Jennifer E. Caselle, Michael Hoban, Dan Malone, Alison Iles

**Affiliations:** 1 Department of Ecology and Evolutionary Biology, University of California Santa Cruz, Santa Cruz, California, United States of America; 2 Department of Integrative Biology, Oregon State University, Corvallis, Oregon, United States of America; 3 Western Ecological Research Center, United States Geological Survey, Santa Cruz, California, United States of America; 4 Marine Science Institute, University of California Santa Barbara, Santa Barbara, California, United States of America; University of California- Santa Barbara, United States of America

## Abstract

Ecological network models and analyses are recognized as valuable tools for understanding the dynamics and resiliency of ecosystems, and for informing ecosystem-based approaches to management. However, few databases exist that can provide the life history, demographic and species interaction information necessary to parameterize ecological network models. Faced with the difficulty of synthesizing the information required to construct models for kelp forest ecosystems along the West Coast of North America, we developed an online database (http://kelpforest.ucsc.edu/) to facilitate the collation and dissemination of such information. Many of the database's attributes are novel yet the structure is applicable and adaptable to other ecosystem modeling efforts. Information for each taxonomic unit includes stage-specific life history, demography, and body-size allometries. Species interactions include trophic, competitive, facilitative, and parasitic forms. Each data entry is temporally and spatially explicit. The online data entry interface allows researchers anywhere to contribute and access information. Quality control is facilitated by attributing each entry to unique contributor identities and source citations. The database has proven useful as an archive of species and ecosystem-specific information in the development of several ecological network models, for informing management actions, and for education purposes (e.g., undergraduate and graduate training). To facilitate adaptation of the database by other researches for other ecosystems, the code and technical details on how to customize this database and apply it to other ecosystems are freely available and located at the following link (https://github.com/kelpforest-cameo/databaseui).

## Introduction

Ecological network models and analyses are recognized for their value in articulating the quantitative and conceptual relationships and emergent properties of natural ecosystems, for generating plausible explanations and testable hypotheses pertaining to community structure and dynamics [1]–[3] and predictions regarding their responses to natural and anthropogenic perturbations [4], [5]. Their importance for informing management and policies has increased markedly with the advent of ecosystem-based management (EBM) approaches (e.g., [6]). EBM requires knowledge of how the human uses of ecosystem services influence the structural (e.g., diversity, composition) and functional (e.g., productivity, nutrient cycling) attributes of ecosystems and how these attributes underpin their integrity and resilience. Quantitative ecosystem models based on species or functional group interaction networks are key tools for understanding how human activities influence ecosystems. These models allow users to forecast how entire ecosystems may respond to alternative management actions. For example, models of species interactions that describe ecosystem-wide effects of anthropogenic perturbations have proven particularly insightful for informing ecosystem-based fisheries management [7], and for understanding the effects of seasonal forcing in freshwater ecosystems [8] and carbon flux in terrestrial forests [9].

However, a critical barrier to the successful implementation of ecosystem-based models is the accessibility of the substantial data they require [10], [11]. An ideal source for this data would be verifiable, comprehensive, relevant, well organized, thoroughly explained, easily updated and readily available at a single location online. Though there is a clear need for accessible online databases tailored for the development of ecological network models, few if any databases meet these criteria. Here, we describe an online interactive database with information (life history, demography, species interactions) required of many ecological network models and that fulfills these and other necessary criteria for expediting the development of these models.

### Why ecological network models need databases

In a comprehensive review of ecological network models used to characterize and explore marine ecosystems, Plagányi [12] identified four general categories of models: Minimum Realistic, Individual Based, Biogeochemical, and Aggregate System Models (Table 1). These four broad categories of ecological network models illustrate the diversity of information that is required of, or can be accommodated by, the various ecological network models. Other model types, such as qualitative loop analysis [13], [14] and allometric trophic network models [8] also benefit from such information. Despite differences in their assumptions and focal applications, all of these modeling approaches accommodate or require some of the same forms of information, such as knowledge of what species, life-stages or functional groups constitute an ecosystem. However, they differ in their requirements or ability to accommodate other forms of information including species' currencies (e.g., biomass, density), distributions, life history or demographic attributes, and the manner in which species interact (e.g., predation, parasitism, competition, mutualism; Table 1). For example, many ecological network models focus entirely on trophic interactions in their representation of species interactions, ignoring non-trophic interactions, such as competition for space [15] or parasitism [16]. The greater the variety of information included in a database, the greater its application across the diversity of ecological network models. Much of the same types of information are also relevant to the development of single-species population models, and are useful in non-modeling contexts. For example, including knowledge of the geographic patterns of species' life history traits and interspecific interactions can help to inform the design of experimental and observational studies, or the placement of marine reserves [17]–[19].

**Table 1:** Comparison and examples of five categories of ecosystem models (modified from Plagany 2007).

Model type & examples	No. of nodes or functional groups	Currency	Life stages	Size structure	Spatial structure	Topology	Diet	Non- trophic interactions	Env. Data
Qualitative models Loop Analysis	<10	Biomass	A	A		Input		A	
Minimum Realistic GADGET^1^	6 to 8	Nutrients	A			Input	R		
Individual Based OSMOSE^2^	7 to 20	Biomass	A	R	A	Input	R	A	
Biogeochemical ATLANTIS	20	Nutrients	A	A	A	Input, output	R	A	R
Aggregate system EwE^3^	>40	Biomass	A		A	Input, output	R	A	A

1 Globally Applicable Area-Disaggregated General Ecosystem Toolbox,

2 Object-oriented simulator of marine ecosystem exploration,

3 Ecopath with Ecosim.

“A” and “R” indicate that the model accommodates or requires information of this form, respectively. “Env” = environmental.

### Shortcomings of existing online databases for ecological network modeling

The diversity of information required by the various kinds of ecological network models is rarely organized in a form that is useful or accessible to modelers. Several well-designed online taxon-specific databases exist that collate information on species taxonomy, phylogeny, life history traits and distribution (Table 2). However, few of these mediate with web browsers or between multiple databases, instead referring to static species-focused summaries. Fewer still translate data requests beyond species-specific searches to permit the querying of multiple species from a common functional group. Having no online database management system (DBMS), these databases preclude the integration of different functions and information in the same process to permit simultaneous access of taxonomic, life history, distribution and ecological databases [20]. Some database management systems (e.g. FishBase, Sea Life Base; Table 2) have the potential to integrate multiple databases in their queries but do not currently do so. Furthermore, database entries do not reference their datum-specific sources, leaving attribution absent or too general and difficult to reconstruct and thereby making validation and reanalysis difficult or impossible.

**Table 2:** List of some of the most relevant marine ecological databases and their attributes to inform ecosystem models.

Database	Subject taxa	Data type	Data visualization	Data export	Integration capabilities
Algae Base	Algae	1,2,5,6,7	x		
All about birds	Birds	1,4,5,6,7	x		
AnAge	Multiple	1,11	x		x
Catalog of Life	Multiple	2	x		x
DataMares	Multiple	2,5,6,9,10	x	x	
EOL	Multiple	1,2,3,4,5,6,7	x		x
Eurobis	Plankton	2,5,6,10	x		x
Fish Base	Fish	1,2,3,4,5,6,7,8	x		x
GoMexi	Multiple	2,8,9,10	x	x	x
ITS	Multiple	2	x	x	x
Kelpforest	Multiple	1,2,3,4,5,6,7,8,9,10,11	x	x	x
Sea Life Base	Multiple	1,2,3,4,5,6,7	x		x
Sea Net	Multiple	1,2,4,5,6,7	x		
Simon	Multiple	1,2,3,4,5,6,7	x		
WoRMS	Multiple	2	x		x

“X” indicates available function. “Integration capability” refers to ability to link with another database.

Data types: 1) Life history, 2) taxonomy, 3) biometrics, 4) behavior, 5) distribution, 6) habitats, 7) photos, 8) species interactions, 9) temporal explicit data, 10) spatial explicit data, 11) references.

Score: Sum of number of attributes valuable for data accessibility for ecosystem modelers. These attributes provide basic information for the parameterization and validation of ecosystem models.

More generally, few existing databases housing information relevant to ecological network models also include information on species interactions. Those that do, include only the presence of the interactions without source citations or detailed description of their nature, spatial, or temporal patterns specific to those interactions. Hence, variation and uncertainties in interaction information are difficult to obtain and remain challenging to incorporate into ecological network models.

### Ecological network models for kelp forest ecosystems

Kelp forests are stands of large macroalgae of the Order Laminariales that occur on temperate and boreal rocky reefs around the world and are among the most productive and diverse ecosystems in the world (reviewed by [21]–[23]). These species-rich ecosystems provide many ecosystem functions, including primary production, habitat for fishes, invertebrates, mammals, and birds, and nurseries for a diversity of species (reviewed by [24]–[27]). Kelp forests also provide humans with many services, including carbon sequestration, shoreline protection and non-consumptive recreational activities [27], [28]. In particular, they support economically and culturally significant commercial and recreational fisheries (e.g., [29], [30]).

Species interactions are known to be key determinants of the structure and dynamics of kelp forests around the word such as the west coast of the United States [22], [24], [27], [31], North Atlantic [32], [33], Mexico [34], Australia and Tasmania [35] yet these are sensitive to anthropogenic and natural perturbations [35]–[37]. Given the importance and complexity of their species interactions, kelp forest ecosystems are strong candidates for ecosystem-based management, which greatly benefits from the use of ecological network models [26].

Only recently, a number of ecological network models have been generated for kelp forests including Espinosa-Romero [38], Ortiz [39], Brynes *et al.* [40] and Marzloff *et al.* [41], for the west coast of Canada, northern Chile, and southern California, respectively. In addition, theoretical multi-species models (not parameterized empirically), have enhanced our understanding of complex interactions in kelp forest systems [42], [43] and assemblages of sessile invertebrates on temperate rocky reefs [44]. Each of these models represents local species composition and, justifiably, over-simplify the networks of kelp forest species interactions. Model-simplification can reflect a compromise between computational power, model-sensitivity, user interests, and preconceptions, but in many cases is simply a result of a lack of accessible information about life history traits and species interactions.

In the process of our development of ecological network models for the kelp forests of the eastern Pacific we found the necessary life history, demographic, and species interaction information poorly synthesized and organized and difficult to access. For these reasons, we developed an online database to collate and freely disseminate information on species life histories, demography, and species interactions. Here, we describe the development of and rationale for the database structure, and the means of accessing the information. Our goal here is to facilitate its use and describe its potential implementation for other ecosystems. That is, although the database was constructed with a focus on kelp forests, the interface, structure, utilities and functions could be easily translated for use in any other ecosystem. Moreover, because the architecture of this database is a DBMS, it can be integrated into a more comprehensive database integrating multiple ecosystems.

## Methods and Results

The overarching goals of the online database, hereafter referred to as the “kelpforest database”, was to create a database management system that could be conveniently populated and utilized across the community of researchers and provide users with the diversity of information required by the various types of ecosystems models. The kelpforest database consists of seven components: 1) a database management system, 2) database homepage, 3) an online data entry interface, 4) an online data entry manual, 5) graphic visualizations, 6) data export tools, and 7) a user forum for discussions, online assistance, and notification of problems. To promote and expedite adaptation of the database for modeling other ecosystems, technical information for developers is readily available, hosted at https://github.com/kelpforest-cameo/databaseui

### Database management system

**Figure 1:**
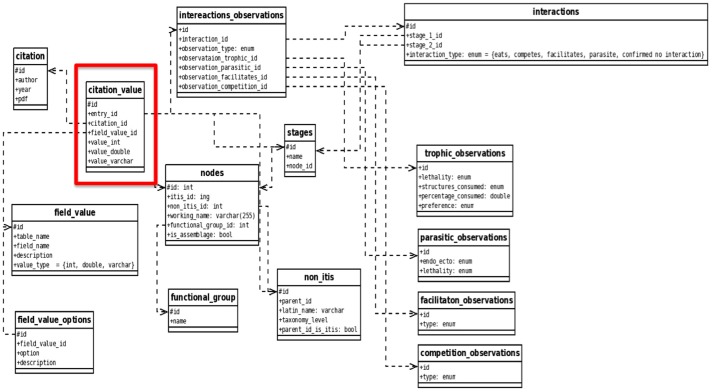
Database schema to enter, query and retrieve data for parameterizing ecological network models. The central element of the schema is the citation value table that links all entries and queries to the data source (red box). The ITIS identifier in the “nodes” table (red line) is used to link the kelpforest database to the ITIS database.

The database is a relational database management system that uses Structured Query Language (MySQL) and Personal Hypertext Preprocessor (PHP) languages and is hosted at the University of California Santa Cruz (http://kelpforest.ucsc.edu/). The central element of the database schema is the source (i.e. citation) of each datum entered (Figure 1). This allows all possible entries and queries to be referenced to the source of that information. This reference avoids redundant entries and promotes quality control by ensuring the legitimacy of entered data. The relational database links the various data tables of the database. Taxonomic information is linked to the Integrated Taxonomic Information System (ITIS; www.ITIS.gov) to ensure that entries are standardized (e.g., avoiding misspellings) and that taxonomic designations and synonyms are continuously updated.

### Database website

The database website is created using WordPress web software (wordpress.org), providing an introduction to the database that includes its purpose, information on how to access it, and up-to-date contact information. The website hosts the other components of the database (i.e., data entry interface, visualization and export tools, user forum), and provides access to a sign-up form for users who wish to obtain data-entry privileges. Access to the data itself does not require registration.

### Online data entry interface

The data entry interface allows multiple users to simultaneously enter information into the database. Access to the data entry interface requires a username and password. This username is linked to every datum entered by an individual in order to provide attribution of user contributions and to simplify quality control. A “sandbox” replica of the database and its data-entry interface allows individuals to practice entering data that will not be archived. Access to this “sandbox” does not require user registration.

The data entry interface provides links to three separate data entry forms: *nodes*, *interactions*, and *citations*. All forms are used to enter and look at information. The *nodes* form is used to enter information relevant to taxa (i.e. species, higher taxonomic units, or species groups). The *interactions* form is used to enter information characterizing interaction between nodes. The *citations* form is to enter the citation information associated with each datum that is entered. Each form contains a range of different sub forms. We therefore, first, provide an overview of each form before detailing its contents.

Within the *nodes* form, the user may list or search for existing nodes, or enter a new node. The first section of the *nodes* form indicates information that is relevant to the entire node, whereas the second section pertains to life stage-specific information. (The database distinguishes between a node's different life stages, detailed below).

The *interactions* form allows users to enter interaction information between specific life stages of two previously entered nodes. Importantly, species interactions are recorded as stage-specific observations of the interaction. That is, multiple observations of an interaction between two focal species (stages) may be recorded from different source citations or from the same citation (pertaining, for example, to different locations or time-periods). We believe such information is key to describing the breadth, spatio-temporal variation, and uncertainty in our knowledge of species interactions.

The *citations* form allows users to enter new citations and authors to which entries are to be attributed, and to list all previously entered authors and full citations. The citation form requires users to identify the category of the source information. That is, sources from which entries have been obtained to-date are primarily from the published peer-reviewed literature, but also include unpublished reports, theses, other online databases, unpublished datasets, and qualified personal observations. The *citation* form is directly linked to the *nodes* and *interactions* forms. Every entry requires a citation. Check boxes located next to each source citation on the list of entered source citations permit data-entry users to indicate when all its pertinent information has been extracted.

### Data entry fields and manual

**Figure 2:**
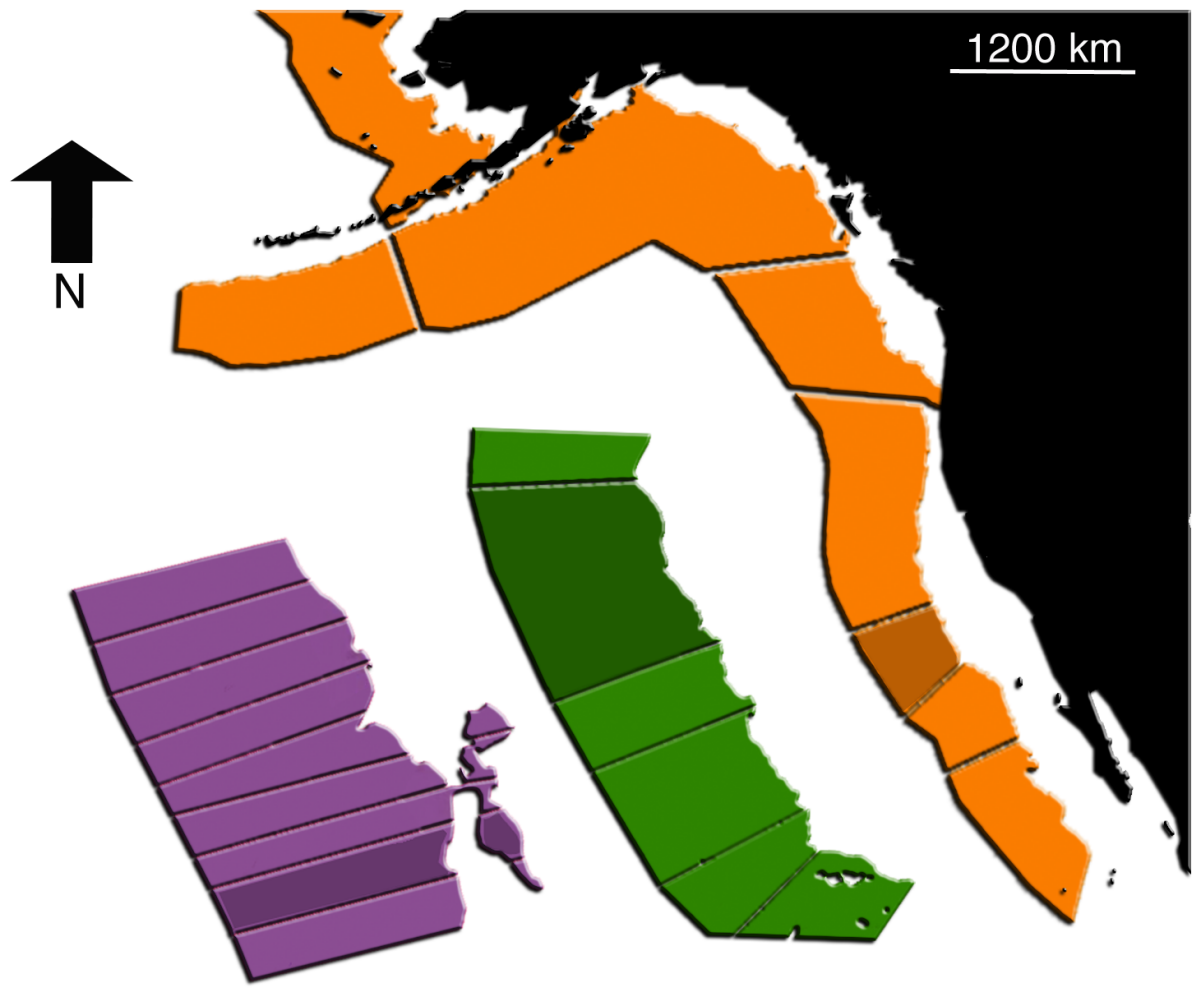
Map interface with delimited regions in the northeast Pacific. The interface allows users to identify location from which data were collected in the data entry process. Orange color represents regions, green sub regions and purple locations.

All entry fields in both the *nodes* and *interactions* forms permit inclusion of the temporal and geographic information associated which each entry. The “time stamp” sub form for individual entry fields allows users to specify whether an entry pertains to a single time point or a window of time points at daily to annual scales. Nodes and their stage-specific interactions may be specified with a geographic location, or range of locations. Location(s) can be identified using either a map-based interactive interface or by entering a latitude and longitude. Nodes and interaction observations are thus geo-referenced across a range of spatial resolutions spanning regional, sub regional, and within sub regional scales and point locations (Figure 2). Regions and sub regions are based on recognized biogeographic sections of the eastern Pacific coast spanning from Baja, Mexico to the western Aleutian Islands. Polygons within each sub region reflect 20 km sections of the coast. Each of these standardized spatial units can be identified by the user directly on the map, or from a hierarchical legend in the mapping interface.

Each entry field also includes a comment box that allows users to clarify their input, when necessary. This is a critical element of the database. Many variables required by ecological network models are not directly available in the literature and must be calculated. The comment box allows users to describe the equations or methods that were used to derive values or standardize units from the information that was available in a given source. For example, estimates of biomass density are often derived from estimates of population size structure and density.

Data entry and standardization is facilitated by drop down menus and “mouse over” descriptions of each data entry field. In addition, the online data entry manual provides users with an overview of the database schema and the interface forms, as well as general information on data entry protocols, tips, and shortcuts.

### Content

As introduced above, there are two general categories of content that may directly or indirectly inform kelp forest ecological network models: content associated with the characterization of nodes, and content describing observations of between–node interactions.

#### Nodes

We refer to the basic taxonomic units of the database as “nodes” rather than “species” or “taxon” because these may represent differing taxonomic resolutions (species, genera, family, etc.), or aggregated assemblages of indistinguishable taxa (e.g., phytoplankton). Each node is identified with a unique node identification number (nodeID), a common or “*working name*”, scientific name, and is associated with an Integrated Taxonomic Information System (ITIS) identifier number. ITIS is an international partnership (USA, Canada and Mexico) that provides consistent and reliable information on the taxonomy and nomenclature of species in North America. Integration with the ITIS database allows nodes to be organized in a current taxonomic hierarchy and minimizes errors associated with relic synonyms and misspelled taxon names. However, the ITIS database is not complete, some taxa or assemblages found along the eastern Pacific are absent. Our database stores these nodes separately, identifying them using the working name and the ITIS id of its most resolved taxonomic level until they become available in ITIS.

Characterization of a node includes life history traits (e.g., reproductive strategy, age and size at maturity, maximum body size) and demographic information (e.g., production–biomass ratios, consumption-biomass ratios, length-weight relationships, von Bertalanffy equations, biomass). This information maybe specific to the ontogenetic stages of a node, or specified as “general” when stage-specificity is unknown. The number and types of stages may be customized for each node, with users choosing from an open-ended list of potential stages when stage-specific information is to be entered. Currently, animal stages include egg, larvae, juvenile, adult, and dead. Algae stages include sporophyte, gametophyte, and dead.

The database was initially populated with species lists from the Partnership for Interdisciplinary Studies of Coastal Oceans (PISCO– www.piscoweb.org), Reef Check California (http://reefcheck.org/rcca/rcca_home.php), Cailliet's *et al.* [45], the Monterey Bay National Marine Sanctuary Integrated Monitoring Network (SIMoN- http://sanctuarysimon.org/) and a species interaction table created by Byrnes *et al.* [46]. Many other species have since been included as a result of an intensive literature search.

#### Interactions

Four general categories of interactions between nodes are included in the database: trophic, competitive, facilitative, and parasitic. Individual observations for all of these interaction categories are described by their observation type (e.g., direct observation, diet analysis) and must be attributed to a source citation. Each interaction category also has entry fields particular to it. For example, trophic interactions may be described by their lethality, the structures consumed, and the percent of the consumer's diet that a particular resource represents. Similarly, parasitic interactions may be described as being endo- or ectoparasite, and by their prevalence and intensity. The interactions between two nodes are not assumed to be reciprocal.

#### Citations

Though most information in the database will likely continue to be extracted from the published, peer-reviewed literature, the demand for information with which to inform modeling efforts motivates a means for making it available that is faster than the rate at which it can be published. Thus, to accommodate unpublished data and personal observations, citations may refer to individuals who provide their contact information.

### Data visualization

**Figure 3:**
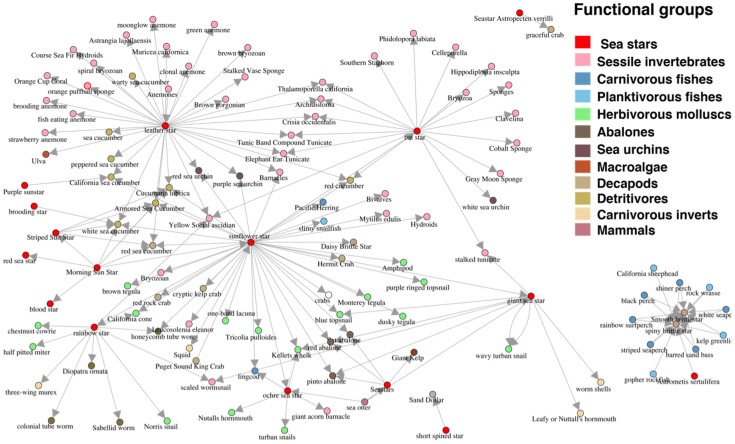
Example of the database visualization tool illustrating a trophic interaction network for an assemblage of kelp forest seastars, color-coded by functional group after Graham *et al.* [25].

A series of static and dynamic visualization tools permit real-time access and interaction with the information contained in the database. These tools query the database in real-time to produce graphics (Figure 3) and tables of summary statistics, interaction networks, adjacency matrices, body size frequency distributions, and interaction observation maps. These utilities rely on a combination of PHP and MySQL languages and capitalize on the capabilities of D3.js (http://d3js.org), a JavaScript library that uses Hyper Text Markup Language (HTML), Scalable Vector Graphics (SVG), and Cascading Style Sheets (CSS) to create and manipulate data-driven visualizations.

### Data export

Information in the kelpforest database is public and accessible to unregistered users through several export tools. These include database queries for tables and matrices containing information about nodes, interactions and citations, allowing users to download the data as comma-separated values (CSV) files (Table 3). Future additions will permit registered users to query the database directly.

**Table 3:** Example of a csv file exported from the database.

Node 1 working name	Node 1 Stage	Node 2 working name	Node 1 Stage	Type	Citations
b&y rockfish	general	crabs	general	trophic	(Larson, 1972)
b&y rockfish	general	tubesnout	general	trophic	(Quast, 1968)
b&y rockfish	general	kelp perch	general	trophic	(Robbins, 2006)
b&y rockfish	general	spiral bryozoan	general	trophic	(Perez, 1981)
b&y rockfish	general	foliate kelp crab	general	trophic	(Hines, 1982)
b&y rockfish	general	moss crab	general	trophic	(Hines, 1982)
b&y rockfish	general	sharpnose crab	general	trophic	(Hines, 1982)
b&y rockfish	general	cryptic kelp crab	general	trophic	(Hines, 1982)
b&y rockfish	general	hairy chiton	general	trophic	(Love et al. 2002)
b&y rockfish	general	monkeyface prickleback	general	trophic	(Love et al. 2002)
b&y rockfish	general	rock prickleback	general	trophic	(Love et al. 2002)
b&y rockfish	general	giant kelpfish	general	trophic	(Love et al. 2002)
b&y rockfish	larval	copepods	general	trophic	(Love et al. 2002)
b&y rockfish	juvenile	zooplankton	general	trophic	(Love et al. 2002)
b&y rockfish	adult	monkeyface prickleback	general	trophic	(Love et al. 2002)
b&y rockfish	adult	rock prickleback	general	trophic	(Love et al. 2002)
b&y rockfish	adult	northern clingfish	general	trophic	(Love et al. 2002)
b&y rockfish	adult	rockweed gunnel	general	trophic	(Love et al. 2002)
b&y rockfish	adult	fluffy sculpin	general	trophic	(Love et al. 2002)
b&y rockfish	adult	smoothhead sculpin	general	trophic	(Love et al. 2002)
b&y rockfish	adult	striped kelpfish	general	trophic	(Love et al. 2002)
b&y rockfish	adult	octopus	general	trophic	(Love et al. 2002)
b&y rockfish	adult	amphipods	general	trophic	(Love et al. 2002)
b&y rockfish	adult	polycheate worm	general	trophic	(Love et al. 2002)
b&y rockfish	general	china rockfish	juvenile	trophic	(Love et al. 2002)
b&y rockfish	general	squid	general	trophic	(Love et al. 2002)
b&y rockfish	general	octopus	general	trophic	(Hallacher & Roberts, 1985)
b&y rockfish	general	blue rockfishes	juvenile	trophic	(Hallacher & Roberts, 1985)
b&y rockfish	general	euphausiids	general	trophic	(Hallacher & Roberts, 1985)
b&y rockfish	general	caprellid amphipod	general	trophic	(Hallacher & Roberts, 1985)
b&y rockfish	general	gammarid amphipod	general	trophic	(Love et al. 2002)
b&y rockfish	general	Idotea isopods	general	trophic	(Hallacher & Roberts, 1985)
b&y rockfish	general	polycheate worm	general	trophic	(Hallacher & Roberts, 1985)
b&y rockfish	general	hydroids	general	trophic	(Hallacher & Roberts, 1985)

This table identifies some trophic interactions of the black and yellow (b&y) rockfish (Sebastes chrysomelas).

## Discussion

Our overarching goal in developing the kelpforest database is to provide a means for expediting the process by which information is accumulated, organized, and made accessible to those making and using ecological network models specific to temperate kelp forests. Its development has been greatly facilitated by collaborations involving federal agency scientists and academics from Canada, the United States, and Mexico. As such, we believe that with similar collaborations, its framework is applicable to any ecosystem. Our description of the structure and elements of the database is meant to inform the reader of the system's capabilities, to both motivate interest in contributing to and using the information it contains, and to suggest features to consider in the development of other databases.

In our experience to date, the online presence of the kelpforest database has been one of its most important features, allowing the research community to populate and access the database simultaneously and internationally. This has greatly enhanced the rate at which the database has been populated with entries and has facilitated communication among the kelp forest research community. To date, 81 registered users across 7 institutions, the majority of whom are undergraduate and graduate students, have contributed to populating the database. Thus, this database has been used as an education and training tool for human resources from different backgrounds. Through their combined effort, the database currently contains 795 nodes and 3616 interactions based on 515 citations. That said, a critical component of the database's online nature is also the online support provided to users through the online forum, webpage, manual, and data field features described above.

A second key feature adding value to the database has been its ability to accommodate a variety of data sources, including information from the literature and existing databases, as well as user-generated values (including our own field data collection to actively fill data gaps identified by the database) and values calculated by synthesis of data in the peer-reviewed and grey literature. This has both enabled users to populate the database with their own information demands, and has made the same information immediately available to other users. Thus, the database is a clearinghouse of information on species life histories, demography and species interactions that are useful not only in the development of kelp forest ecological network models, but also for a variety of other ecological applications. The database has thereby served to inform the design of observational and experimental studies at our institutions; it has been used to train students in the use and applications of this tool, and promoted collaboration between research institutions.

Of course, few if any databases will ever collect all the relevant knowledge that has and is being obtained about kelp forest ecosystems. Databases need to be sufficiently flexible to not only accommodate new information as it is generated, but also to accommodate new kinds of information. For examples, as genetic information becomes increasingly available, the database could be modified to integrate this new information and enable users to explore the genetic basis of varying demographic relationships and species interactions and how variation in those variables contribute to patterns of genetic variability and structure and ecological-evolutionary feedbacks. To facilitate the expansion and evolution of this database and its adoption for other ecosystem databases, access to the code and technical details on how to customize this database and apply it to other ecosystems is freely available and located at the following link (https://github.com/kelpforest-cameo/databaseui).

We see the development of the kelpforest database as an important step forward toward a simpler, more organized, and more reliable integration of the collective biological knowledge of species life histories, demographics, and interactions. Our goal is to enhance the accessibility and quality of information in order to facilitate the development and use of ecological network models and inform ecosystem-based approaches to management.

## References

[1] Montoya JM, Pimm SL, Solé RV (2006) Ecological networks and their fragility. Nature 442: 259–264. 16855581 10.1038/nature04927

[2] Thompson RM, Brose U, Dunne JA, Hall RO, Hladyz S, et al (2012) Food webs: reconciling the structure and function of biodiversity. Trends Ecol Evol 27: 689–697. 22959162 10.1016/j.tree.2012.08.005

[3] Borrett SR, Moody J, Edelmann A (2014) The rise of Network Ecology: Maps of the topic diversity and scientific collaboration. Ecol Modell 7158: 1–17.

[4] Clark JS, Carpenter SR, Barber M, Collins S, Dobson A, et al (2001) Ecological forecasts: an emerging imperative. Science 293: 657–660. 11474103 10.1126/science.293.5530.657

[5] Yodzis P (2001) Must top predators be culled for the sake of fisheries? Trends Ecol Evol 16: 78–84. 11165705 10.1016/s0169-5347(00)02062-0

[6] Fulton E, Link JS, Kaplan IC, Savina-Rolland M, Johnson P, et al (2011) Lessons in modeling and management of marine ecosystems: the Atlantis experience. Fish Fish 12: 171–188.

[7] Ainsworth CH, Morzaria-Luna HN, Kaplan IC, Levin PS, Fulton Ea (2012) Full compliance with harvest regulations yields ecological benefits: Northern Gulf of California case study. J Appl Ecol 49: 63–72.

[8] Boit A, Martinez ND, Williams RJ, Gaedke U (2012) Mechanistic theory and modelling of complex food-web dynamics in Lake Constance. Ecol Lett 15: 594–602. 22513046 10.1111/j.1461-0248.2012.01777.x

[9] Morales P, Sykes MT, Prentice IC, Smith P, Smith B, et al (2005) Comparing and evaluating process-based ecosystem model predictions of carbon and water fluxes in major European forest biomes. Glob Chang Biol 11: 2211–2233. 10.1111/j.1365-2486.2005.01036.x 34991276

[10] Tallis H, Levin PS, Ruckelshaus M, Lester SE, McLeod KL, et al (2010) The many faces of ecosystem-based management: Making the process work today in real places. Mar Policy 34: 340–348.

[11] Hudson L, Reuman D (2013) A cure for the plague of parameters: constraining models of complex population dynamics with allometries. Proc R Soc Biol 19. 10.1098/rspb.2013.1901 PMC3779337 24026824

[12] Plagányi E (2007) Models for an ecosystem approach to fisheries. FAO Fish Tech Pap 477: 107.

[13] Levins R (1974) The qualitative analysis of partially specified systems. Ann N Y Acad Sci 231. 10.1111/j.1749-6632.1974.tb20562.x 4522890

[14] Dambacher JM, Gaughan DJ, Rochet MJ, Rossignol PA, Trenkel VM (2009) Qualitative modeling and indicators of exploited ecosystems. Fish Fish 10: 305–322.

[15] Wootton JT (2001) Local interactions predict large-scale pattern in empirically derived cellular automata. Nature 413: 841–844. 11677606 10.1038/35101595

[16] Lafferty KD, Allesina S, Arim M, Briggs CJ, De Leo G, et al (2008) Parasites in food webs: the ultimate missing links. Ecol Lett 11: 533–546. 18462196 10.1111/j.1461-0248.2008.01174.x PMC2408649

[17] Gerber L, Botsford L, Hastings A, Possingham HP, Gaines SD, et al (2003) Population models for marine reserve design: a retrospective and prospective synthesis. Ecol Appl 13: 47–64.

[18] Micheli F, Halpern B, Botsford LW, Warner RR (2004) Trajectories and correlates of community change in no-take marine reserves. Ecol Appl 14: 1709–1723.

[19] Caselle JE, Hamilton SL, Schroeder DM, Love MS, Standish JD, et al (2011) Geographic variation in density, demography, and life history traits of a harvested, sex-changing, temperate reef fish. Can J Fish Aquat Sci 68: 288–303.

[20] Stein LD (2003) Integrating biological databases. Nat Rev Genet 4: 337–345. 12728276 10.1038/nrg1065

[21] Mann KH (1973) Seaweeds: Their Productivity and Strategy for Growth: The role of large marine algae in coastal productivity is far more important than has been suspected. Science 182: 975–981. 17833778 10.1126/science.182.4116.975

[22] Dayton PK (1985) Ecology of Kelp Communities. Annu Rev Ecol Syst 16: 215–245.

[23] Schiel DR, Foster MS (*in press*) The Biology and Ecology of Giant Kelp Forests. UC press.

[24] Schiel DR, Foster MS (1986) The structure of subtidal algal stands in temperate waters. Oceanogr Mar Biol Annu Rev 24: 265–307.

[25] Graham M, Halpern B, Carr MH (2008) Diversity and Dynamics of Californian Subtidal Kelp Forests. In: McClanahan TR, Branch GM, editors. Food Webs and the Dynamics of Marine Reefs . Oxford: Oxford University Press. pp. 103–134.

[26] Springer Y, Hays C, Carr MH, Mackey M (2010) Toward ecosystem-based management of marine macroalgae—The bull kelp, *Nereocystis luetkeana* . Oceanogr Mar Biol Annu Rev 48: 1–42.

[27] Carr MH, Reed DC (in press) Shallow rocky reefs and kelp forests. In: H M, Zabaleta E, editors. Ecosystems of California. Berkeley: UC Press.

[28] Hölker F, Beare D, Dörner H, di Natale A, Rätz HJ, et al (2007) Comment on “Impacts of biodiversity loss on ocean ecosystem services”. Science 316: 1285 author reply 1285 10.1126/science.1139114 17540886

[29] Starr R, Cope JM, Kerr LA (2002) Trends in fisheries and fishery resources associated with the Monterey Bay National Marine Sanctuary from 1981–2000. San Diego, La Jolla, California.

[30] Ling S, Johnson C (2009) Supplemental material -Overfishing reduces resilience of kelp beds to climate driven catastrophic shift. Proc Natl Acad Sci 1–3. 10.1073/pnas.0907529106 PMC2793314 20018706

[31] Estes J, Palmisano J (1974) Sea Otters: Their Role in structuring nearshore communities. Science 185 4156: 1058–1060. 17738247 10.1126/science.185.4156.1058

[32] Johnson C, Mann K (1988) Diversity, patterns of adaptation, and stability of Nova Scotian kelp beds. Ecol Monogr 58: 129–154.

[33] Steneck RS, Graham MH, Bourque BJ, Corbett D, Erlandson JM, et al (2003) Kelp forest ecosystems: biodiversity, stability, resilience and future. Environ Conserv 29: 436–459.

[34] Beas-Luna R, Ladah LB (2014) Latitudinal, seasonal, and small-scale spatial differences of the giant kelp, Macrocystis pyrifera, and an herbivore at their southern range limit in the northern hemisphere. Bot Mar 57: 73–83.

[35] Ling SD, Johnson CR, Frusher SD, Ridgway KR (2009) Overfishing reduces resilience of kelp beds to climate-driven catastrophic phase shift. Proc Natl Acad Sci U S A 106: 22341–22345. 20018706 10.1073/pnas.0907529106 PMC2793314

[36] Ebeling AW, Laur DR, Rowley R (1985) Severe storm disturbances and reversal of community structure in a southern California kelp forest. Mar Biol 84: 287–294.

[37] Reed DC, Rassweiler A, Carr MH, Cavanaugh KC, Malone DP, et al (2011) Wave disturbance overwhelms top-down and bottom-up control of primary production in California kelp forests. Ecology 92: 2108–2116. 22164835 10.1890/11-0377.1

[38] Espinosa-Romero MJ (2010) Towards ecosystem based management: Integrating stakeholder values in decision making and improving the representation of ecosystems in ecosystem models. Master thesis by The University of British Columbia.

[39] Ortiz M, Avendaño M (2010) Trhopic mass balanced models and dynamic simulations of benthic communities from la Rinconada Marine Reserve off northern Chile: network properties and multispecies harvest scenario assessments. Aquat Conserv Mar Freshw Ecosyst 20: 58–73.

[40] Byrnes JE, Reed DC, Cardinale BJ, Cavanaugh KC, Holbrook SJ, et al (2011) Climate-driven increases in storm frequency simplify kelp forest food webs. Glob Chang Biol 17: 2513–2524.

[41] Marzloff MP, Johnson CR, Little LR, Soulié J-C, Ling SD, et al (2013) Sensitivity analysis and pattern-oriented validation of TRITON, a model with alternative community states: Insights on temperate rocky reefs dynamics. Ecol Modell 258: 16–32.

[42] Baskett ML, Salomon AK (2010) Recruitment facilitation can drive alternative states on temperate reefs. Ecology 91: 1763–1773. 20583717 10.1890/09-0515.1

[43] Marzloff MP, Dambacher JM, Johnson CR, Little LR, Frusher SD (2011) Exploring alternative states in ecological systems with a qualitative analysis of community feedback. Ecol Modell 222: 2651–2662.

[44] Dunstan PK, Johnson CR (2006) Linking richness, community variability, and invasion resistance with patch size. Ecology 87: 2842–2850. 17168028 10.1890/0012-9658(2006)87[2842:lrcvai]2.0.co;2

[45] Cailliet G, Burton E, Cope J, Kerr L, Lartson R, et al.. (2000) Biological characteristics of nearshore fishes of California: A review of existing knowledge and proposed additional studies for the Pacific Ocean Interjurisdictional Fisheries Management Plan Coordination and Development Project.

[46] Byrnes JE, Reed DC, Cardinale BJ, Cavanaugh KC, Holbrook SJ, et al (2011) Climate-driven increases in storm frequency simplify kelp forest food webs. Glob Chang Biol 17: 2513–2524.

